# Friend or Foe: Draft Genome Sequence of *Bradyrhizobium* sp. Strain SRS-191

**DOI:** 10.1128/mra.00753-22

**Published:** 2022-10-10

**Authors:** Ashvini Chauhan, Rajesh Singh Rathore, Meenakshi Agarwal, Adi J. Chauhan, Aryan Taywade

**Affiliations:** a School of the Environment, Florida A&M University, Tallahassee, Florida, USA; b Center for Viticulture and Small Fruit Research, Florida A&M University, Tallahassee, Florida, USA; Indiana University, Bloomington

## Abstract

We report the genomic features of *Bradyrhizobium* sp. strain SRS-191, which was isolated from a former nuclear legacy site in Aiken, South Carolina, USA. With a genome size of 7,621,400 bp, the strain harbored genes not only for environmentally beneficial traits (e.g., heavy metal resistance, nitrogen fixation, and aromatic biodegradation) but also for antimicrobial resistance.

## ANNOUNCEMENT

Environmental microorganisms perform several ecosystem services, including biogeochemical cycling, bioremediation, and plant growth-promoting activities (1). Previous findings, including ours, from the Savannah River Site (SRS) (a former nuclear legacy site in the United States) revealed a dominance of *Bradyrhizobium* species, with a myriad of bioremediative traits (2–4). The long-term heavy metal (e.g., uranium [U]) contamination at the SRS (5) continues to potentially drive evolution of both metal resistance genes (MRGs) (2–4) and multidrug resistance (MDR) genes within the native microbiota. Such environmentally evolved MDR “superbugs” appear to be at a tipping point (6) of causing large-scale human deaths, replacing cancer as the number one cause of human death by the year 2050. To garner a better understanding of these compounding issues, several microbial isolates from SRS soils were obtained (3); strain SRS-191 was chosen for this genomic study on the basis of its heavy metal resistance phenotype. Briefly, SRS soils from the Steed Pond-Tims Branch location were serially diluted and plated on a *Bradyrhizobium*-selective growth medium (3, 7) amended with uranium nitrate hexahydrate; U is the main SRS heavy metal contaminant. Colonies appearing within 1 week at 30°C, under aerobic conditions, were isolated on LB agar, and MIC analysis confirmed the U resistance of strain SRS-191 (3).

**FIG 1:**
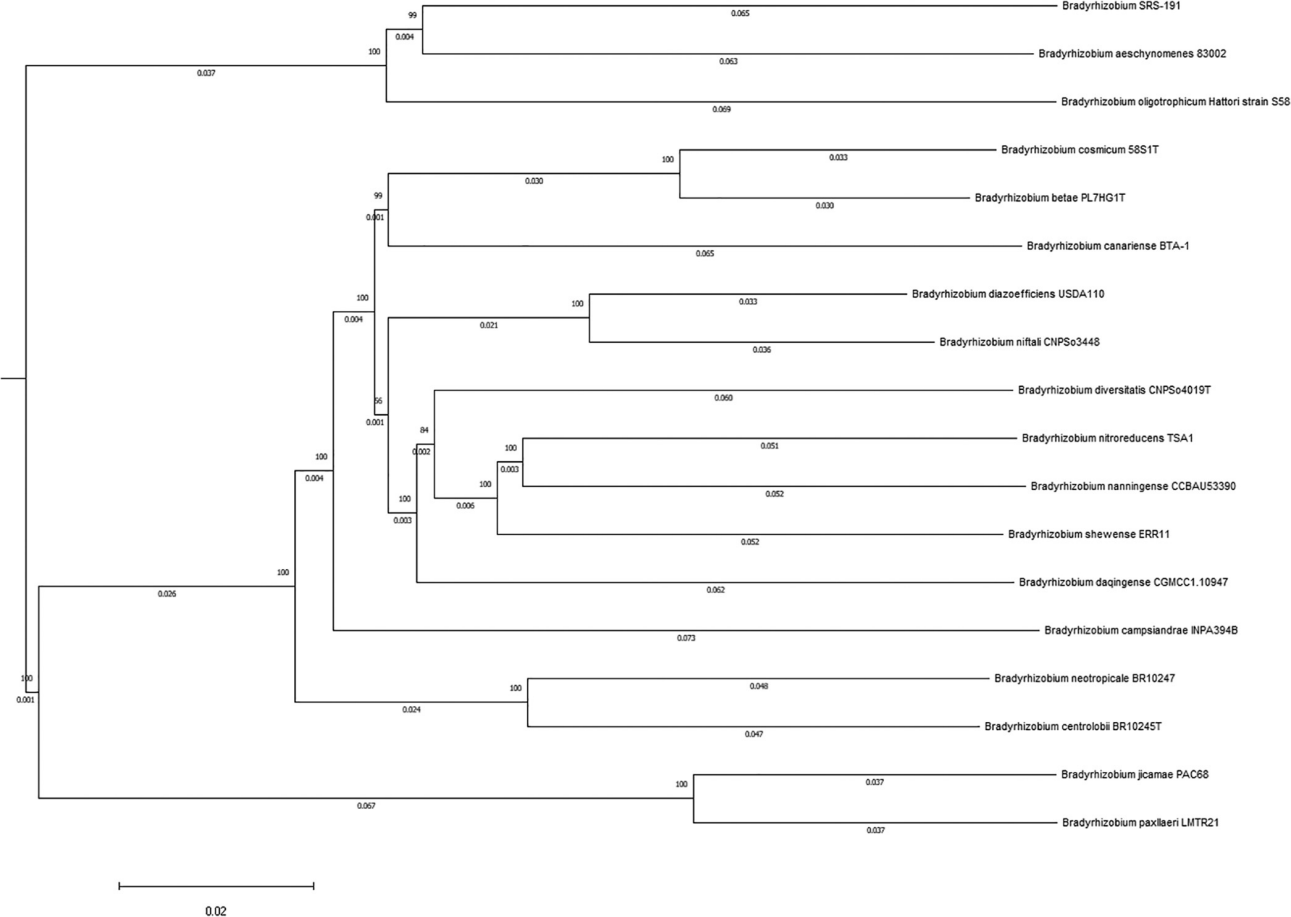
Phylogenomic tree of strain SRS-191, inferred from genome BLAST distance phylogeny (GBDP) distances analyzed using the TYGS pipeline (https://tygs.dsmz.de). The tree was visualized and modified for publication using the MEGA v11 workflow (15). The branch lengths are scaled in terms of GBDP distance formula d5, and the numbers above the branches are GBDP pseudo-bootstrap support values of >60% from 100 replications, with average branch support of 95.9%; the tree was rooted at the midpoint.

Genomic DNA from SRS-191 was extracted using a soil DNA extraction kit (Zymo Research, Irvine, CA, USA) according to the manufacturer’s instructions and quantified using a NanoDrop ND-1000 spectrophotometer. Approximately 300 ng DNA was used to prepare libraries with the NEBNext Ultra II DNA library preparation kit, and the libraries were sequenced with a MiSeq 500-cycle v2 kit (Illumina, San Diego, CA, USA). A total of 820,820 reads, with lengths of 21 to 251 bp, were obtained. Default parameters were used for all bioinformatic analyses except where otherwise noted. Trimming and quality control of obtained reads were performed using Trim Galore (https://www.bioinformatics.babraham.ac.uk/projects/trim_galore). *De novo* assembly was then performed using Unicycler v0.4.8 (8), and the assembly was evaluated using QUAST v5.0.2 (9). The genome of SRS-191 was assembled in 96 contigs, with an *N*_50_ value of 349,972 bases, a size of 7,621,400 bp, and an average GC content of 65.54%. Based on Type Strain Genome Server (TYGS) analysis (10), strain SRS-191 is a potentially new *Bradyrhizobium* species (Fig. 1).

Genome annotation was then performed using the Pathosystems Resource Integration Center (PATRIC) (11) and Rapid Annotation using Subsystems Technology (RAST) (12), which revealed 289 to 355 subsystems (13), coding sequence (CDSs) for 7,104 proteins, 3 rRNAs, and 51 tRNAs. The main subsystems were metabolism (98 subsystems), protein processing (41 subsystems), energy (37 subsystems), stress response-defense-virulence (31 subsystems), and membrane transport (22 subsystems). Interestingly, the strain harbored MRGs (e.g., genes for the cobalt-zinc-cadmium resistance proteins CzcR and CzcD), as well as other environmentally beneficial genes, including genes for nitrogen fixation and biodegradation. However, it was concerning to find 56 MDR genes in SRS-191, a trait identified in several other microbes isolated from SRS soils (2–4, 14). It remains to be determined whether strains such as SRS-191 are among our beneficial microbial friends or our pathogenic foes, given their possession of both MRGs and MDR traits.

### Data availability.

This whole-genome shotgun project has been deposited in NCBI GenBank under the BioSample, BioProject, and SRA accession numbers SAMN29512481, PRJNA855978, and SRR20731430, respectively.
